# Co-infections with Chikungunya Virus and Dengue Virus in Delhi, India

**DOI:** 10.3201/eid1507.080638

**Published:** 2009-07

**Authors:** Harendra S. Chahar, Preeti Bharaj, Lalit Dar, Randeep Guleria, Sushil K. Kabra, Shobha Broor

**Affiliations:** All India Institute of Medical Sciences, New Delhi, India

**Keywords:** Chikungunya virus, dengue virus, viruses, RT-PCR, India, outbreak, co-infections, Aedes aegypti, hemorrhagic manifestations, dispatch

## Abstract

*Aedes aegypti* mosquitoes are common vectors for dengue virus and chikungunya virus. In areas where both viruses cocirculate, they can be transmitted together. During a dengue outbreak in Delhi in 2006, 17 of 69 serum samples were positive for chikungunya virus by reverse transcription–PCR; 6 samples were positive for both viruses.

Chikungunya virus (CHIKV) was isolated in Tanganyika (now Tanzania) in 1953 (*1*). In Asia, this virus is transmitted almost exclusively by *Aedes aegypti* mosquitoes. India had its first CHIKV outbreak in 1963; it was followed by epidemics in other parts of the country (*2*). Recently, massive outbreaks of CHIKV have been reported from many islands in the Indian Ocean (*3*). Chikungunya outbreaks in India were reported in 2005, and 1.4 million chikungunya cases were reported from different states (*3*).

Estimated annual incidence of disease caused by dengue virus (DENV) is 50–100 million cases of dengue fever and 250,000 cases of dengue hemorrhagic fever; mortality rate is 25,000 per year in tropical and subtropical countries. Like CHIKV, DENV is also transmitted by *Ae*. *aegypti* and is endemic to urban and semiurban areas of India (*4*).

In Asia, the CHIKV-affected areas overlap with DENV-endemic areas (*5*,*6*) and provide opportunities for mosquitoes to become infected with both viruses. Co-infection with 2 dengue viruses (DENV-1 and DENV-4) was reported in Puerto Rico in 1982 (*7*). Since then, many cases of concurrent infections with multiple DENV serotypes have been reported in many countries. Since 2005, co-infections with >2 DENV serotypes have been reported in Delhi, India (*8*). Co-infections with DENV and CHIKV were reported in Calcutta, India, in 1967 (*5*). Subsequent serologic investigations in southern India indicated that the 2 viruses can coexist in the same host (*9*). We report detection by reverse transcription–PCR of co-infections with CHIKV and DENV in clinical samples obtained during the 2006 dengue outbreak in Delhi, India.

## The Study

Acute-phase blood samples were collected from 69 patients with clinically suspected cases of DENV/CHIKV co-infection. Viral RNA was extracted from serum samples by using the MagNA Pure Compact Nucleic Acid Isolation System (Roche Diagnostics, Basel, Switzerland). Published primers and cycling conditions were used for the amplification of DENV (*10*) and CHIKV (*11*). cDNA was synthesized by using avian myeloblastosis virus reverse transcriptase (Promega Corp., Madison, WI, USA) and downstream consensus primer (D2) for DENV and random hexamers for CHIKV. DENV typing was performed by using second-round amplification with type-specific primers (*10*). The amplified products were visualized by electrophoresis on 2% agarose gels. Because samples were received during a dengue outbreak, ethical clearance was not required.

Partial nucleotide sequences of the envelope 1 (E1) gene (294 bp) of CHIKV were determined by using an automated 310 DNA sequencer (Applied Biosystems Inc., Foster City, CA, USA). Sequences were aligned, analyzed, subjected to homology search by BLAST analysis (www.ncbi.nlm.nih.gov/Education/BLASTinfo/information3.html), and submitted to GenBank (accession nos. EU727159–63 and EF539265). Phylogenetic analysis of CHIKV sequences (Table) was conducted by using ClustalW (www.ebi.ac.uk/Tools/clustalw2/index.html) and MEGA version 3.1 software (*12*), Kimura 2-parameter distances, and neighbor-joining algorithms.

**Table:** Chikungunya virus sequences, including strains from Delhi and southern India, used for phylogenetic analysis*

Sequence no.	Laboratory ID or isolate name	Year	State/country	GenBank accession no.
1	GOA 018	2006	Goa/India	EF187902
2	HYD 349	2006	Hyderabad/India	EF187893
3	GWL 008	2006	Madhya Pradesh/India	EF187904
4	HYR023	2006	Karnataka/India	EF187899
5	CHTR 54	2006	Andhra Pradesh/India	EF187897
6	IND06 AP5	2006	Andhra Pradesh/India	DQ520744
7	IndKL 01	2006	Kerala/India	EU119154
8	IND06 MH1	2006	Maharashtra/India	DQ520734
9	IND06 AP6	2006	Andhra Pradesh/India	DQ520745
10	IND06 MS2	2006	Andhra Pradesh/India	DQ520740
11	IND06 MS1	2006	Andhra Pradesh/India	DQ520741
12	IND06 KA3	2006	Karnataka/India	DQ520738
13	PON1	2006	Pondicherry/India	EF113095
14	IND05 KA1	2005	Karnataka/India	DQ520737
15	REUNION	2006	Réunion Island	DQ443544
16	IND06 AP3	2006	Andhra Pradesh/India	EF027134
17	IND06 MH2	2006	Maharashtra/India	EF027136
18	IND06 MH3	2006	Maharashtra/India	DQ520736
19	ROSS	1953	Tanzania	AF490259
20	TAN53	1953	Tanzania	AF192905
21	IND00 MH4	2000	Maharashtra/India	EF027139
22	CONGO02	2000	Congo	AY549581
23	CONGO03	2000	Congo	AY549579
24	CONGO01	2000	Congo	AY549583
25	S27AFRICA	1953	Tanzania	NC004162
26	MALAYA98A	1998	Malaysia	AF394210
27	MALAYA98B	1998	Malaysia	AF394211
28	THAI95	1995	Thailand	AF192897
29	THAI96	1996	Thailand	AF192900
30	THAI88	1988	Thailand	AF192896
31	PHILLIP85	1985	The Philippines	AF192895
32	INDON85	1985	Indonesia	AF192894
33	THAI75	1975	Thailand	AF192898
34	THAI78	1978	Thailand	AF192899
35	THAI62	1962	Thailand	AF192908
36	IND71CH1	1971	Tamil Nadu/India	DQ520751
37	IND63WB1	1963	West Bengal/India	EF027140
38	IND64CH2	1964	Tamil Nadu/India	DQ520748
39	SENEG66	1966	Senegal	AF192891
40	NIGER64	1963	Nigeria	AF192893
41	SENEG83A	1983	Senegal	AY726732
42	SENEG83B	1983	Senegal	AF192892
43	O’NYONG-NYONG	1996	Uganda	AF079456
44	DEL/1467/06	2006	Delhi/India	EF539265
45	DEL/758/06	2006	Delhi/India	EU727160
46	DEL/868/06	2006	Delhi/India	EU727163
47	DEL/968/06	2006	Delhi/India	EU727161
48	DEL/1307/06	2006	Delhi/India	EU727162
49	DEL/1795/06	2006	Delhi/India	EU727159

*ID, identification number.

**Figure 1:**
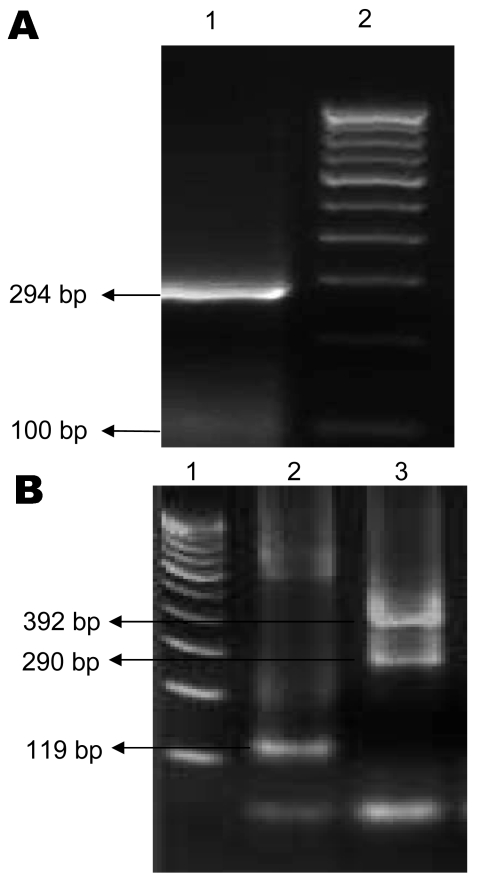
Agarose gel electrophoresis showing chikungunya virus (A) and dengue virus (B) PCR products. A) Lane 1, 294-bp product specific for chikungunya virus; lane 2, 100-bp DNA marker. B) Lane 1, 100-bp DNA marker; lane 2, 119-bp product specific for dengue 2 virus; lane 3, 290-bp product for dengue 3 virus and 392-bp product for dengue 4 virus.

Of 69 samples tested, DENV RNA was detected in 48 and CHIKV RNA in 17. Of the 17 CHIKV-positive samples, 6 were co-infected with DENVs. Three of the 6 samples from patients co-infected with CHIKV/DENV contained DENV-3; 1 contained DENV-4, and 2 contained 2 DENV serotypes (1 contained DENV-3 and DENV-4 and 1 contained DENV-3 and DENV-1) (Figure 1).

Of the 17 CHIKV-positive patients, 10 were male and 6 were female. Information regarding age, sex, and clinical features was not available for 1 patient. Thirteen samples were from adults (>12 years of age) and 3 were from children (<12 years of age).

Retrospective analysis of medical records identified clinical information for 6 patients co-infected with DENV and CHIKV. All 6 patients had fever, headache, joint pain, and low thrombocyte counts (<100,000/mm^3^). The patients with only CHIKV infection had fever, headache, and joint pain. Of the 6 patients with co-infections, 2 had dengue hemorrhagic fever with central nervous system (CNS) involvement. CNS involvement and hemorrhagic manifestations may be caused by DENVs because these manifestations are common in patients infected with DENV; CNS involvement has been documented in persons with DENV infections (*13*). In 2 patients with CNS involvement, 1 was infected with DENV-3 and 1 was infected with DENV-4. Of the 6 patients with co-infections, 5 fully recovered and 1 died.

**Figure 2:**
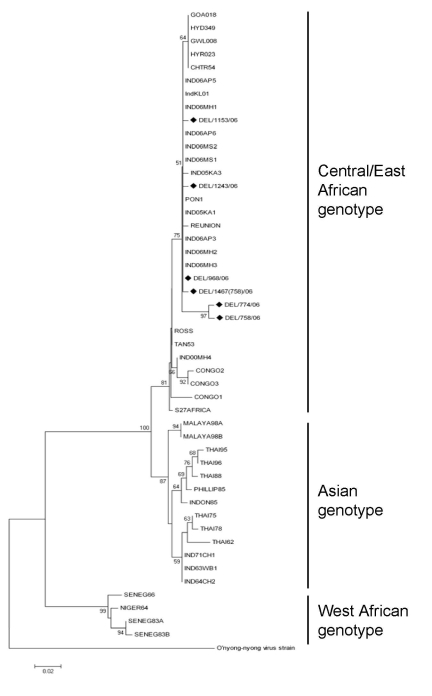
Phylogenetic analysis of partial envelop 1 (E1) gene sequences (294 bp) of chikungunya virus strains from the 2006 dengue outbreak in Delhi, India. Neighbor-joining tree was constructed by using E1 gene sequences from various chikungunya virus sequences. O’nyong-nyong virus (AF079456) was used as an outgroup. Percentage bootstrap support is indicated by the values at each node. Delhi strains are indicated by a diamond. Scale bar indicates nucleotide substitutions per site.

Phylogenetic analysis of partial E1 gene sequences demonstrated that all CHIKV strains from Delhi grouped with isolates obtained during 2006 from southern India and islands in the Indian Ocean and belonged to the Central/East African genotype (Figure 2). This finding indicates that during 2006 similar strains were circulating throughout India. Isolates obtained in India during 1963–1973 clustered with isolates from Thailand (Thai 62–78) and formed a separate cluster in the Asian genotype.

## Conclusions

For many years, it appeared that CHIKV had disappeared from India, but late in 2005 the virus reemerged on Reunion Island and in India (*3*). Confirmed cases of CHIKV infection have been reported from Delhi, Haryana, Uttar Pradesh, and Rajasthan provinces in northern India, although these states did not have large-scale epidemics (*14*).

DENV infections are endemic to northern India; in recent years, increasing trends of cocirculation of multiple DENV serotypes in Delhi suggest that DENVs are becoming hyperendemic to this region (*8*). During 2006, DENV and CHIKV were detected in Delhi (*14*). Because the clinical features of DENV and CHIKV are similar, CHIKV infections may go undiagnosed in DENV-endemic areas. In India, *Ae*. *aegypti* mosquitoes are primary vectors for DENV and CHIKV, and opportunities for co-infections in humans are increased by the feeding behavior of the mosquito (*15*), low socioeconomic conditions, and high population density.

We report co-infections with DENV and CHIKV in India after a long absence of the viruses in this region. It is difficult to comment on increased severity of illness in patients with DENV/CHIKV co-infections because the number of patients tested was small. Additional clinical information is needed to determine the influence of co-infections on clinical expression of dengue and chikungunya fever.

Our study indicates that co-infections with CHIKV and DENV occur in areas where these 2 viruses cocirculate. Concurrent infections may result in illness with overlapping signs and symptoms, making diagnosis and treatment difficult for physicians. Repeated outbreaks of dengue, recent activity of CHIKV, and CHIKV/DENV co-infections in the Delhi area suggest that the epidemiology of these viruses is changing in this region and that these viruses are becoming endemic to this region. Thus, in clinically suspected cases of dengue or chikungunya fever, it is advisable to test for both viruses in areas where they cocirculate.
